# Nasopulmonary mites (Acari: Halarachnidae) as potential vectors of bacterial pathogens, including *Streptococcus phocae*, in marine mammals

**DOI:** 10.1371/journal.pone.0270009

**Published:** 2022-06-16

**Authors:** Risa Pesapane, Andrea Chaves, Janet Foley, Nadia Javeed, Samantha Barnum, Katherine Greenwald, Erin Dodd, Christine Fontaine, Padraig Duignan, Michael Murray, Melissa Miller

**Affiliations:** 1 Department of Veterinary Preventive Medicine, College of Veterinary Medicine, The Ohio State University, Columbus, Ohio, United States of America; 2 School of Environment and Natural Resources, College of Food, Agricultural, and Environmental Science, The Ohio State University, Columbus, Ohio, United States of America; 3 Department of Medicine and Epidemiology, School of Veterinary Medicine, University of California Davis, Davis, California, United States of America; 4 California Department of Fish and Wildlife, Marine Wildlife Veterinary Care and Research Center, Santa Cruz, California, United States of America; 5 The Marine Mammal Center, Sausalito, California, United States of America; 6 Monterey Bay Aquarium, Monterey, California, United States of America; Beni Suef University Faculty of Veterinary Medicine, EGYPT

## Abstract

Nasopulmonary mites (NPMs) of the family Halarachnidae are obligate endoparasites that colonize the respiratory tracts of mammals. NPMs damage surface epithelium resulting in mucosal irritation, respiratory illness, and secondary infection, yet the role of NPMs in facilitating pathogen invasion or dissemination between hosts remains unclear. Using 16S rRNA massively parallel amplicon sequencing of six hypervariable regions (or “16S profiling”), we characterized the bacterial community of NPMs from 4 southern sea otters (*Enhydra lutris nereis)*. This data was paired with detection of a priority pathogen, *Streptococcus phocae*, from NPMs infesting 16 southern sea otters and 9 California sea lions (*Zalophus californianus*) using nested conventional polymerase chain reaction (nPCR). The bacteriome of assessed NPMs was dominated by Mycoplasmataceae and Vibrionaceae, but at least 16 organisms with pathogenic potential were detected as well. Importantly, S. *phocae* was detected in 37% of NPM by nPCR and was also detected by 16S profiling. Detection of multiple organisms with pathogenic potential in or on NPMs suggests they may act as mechanical vectors of bacterial infection for marine mammals.

## Introduction

Nasopulmonary mites (NPMs) of the family Halarachnidae are obligate endoparasites that colonize the respiratory tract of mammals. Two genera with five extant species infest marine mammals: *Orthohalarachne* which primarily infests eared seals (Otariidae) and walrus (Odobenidae), and *Halarachne* which infests earless seals (Phocidae) and sea otters (*Enhydra lutris*) [1–5]. NPMs are viviparous [6,7] so their life cycle only consists of larvae, protonymphs, deutonymphs, and adults [2,6]. The protonymph and deutonymph stages are of such short duration that they are non-feeding and essentially inactive [6]. Adults are similarly non-motile because their well-developed tarsal claws anchor them in place [6]. In contrast, the larval stage is highly motile and hardy outside of the host [2,6] making them largely responsible for host-to-host transmission [6]. Transmission is presumed to occur via direct exchange of larval mites during close contact [6,8], but the persistence of larval mites in saline suggests environmental transmission is also plausible [2,6].

Although sometimes regarded as benign [9], NPMs can cause substantial respiratory pathology in a variety of marine mammal hosts [10–13]. Recent studies by our team concluded that nasopulmonary acariasis is an underappreciated contributor to southern sea otter (*E*. *lutris nereis*) morbidity and mortality, with up to one quarter of fresh dead otters necropsied between 2012 and 2017 harboring NPM [2,13,14]. In a complementary study of sympatric pinnipeds, the prevalence of NPM infestation ranged from 8.8% to 74.1% among pinniped populations [15]. The piercing mouthparts of NPMs damage surface epithelium while consuming bodily fluids [9] resulting in mucosal irritation, respiratory illness, and secondary infection [1,10–13,16]. Despite increasing reports of respiratory infection associated with mite infestation [12,13,16], the role of NPMs in facilitating or disseminating pathogens is unknown.

*Streptococcus phocae* and related beta-hemolytic Streptococcci are opportunistic bacterial pathogens of emerging importance in southern sea otters and other marine species that can invade their hosts through damaged skin or mucosa [17,18]. Infection with *S*. *phocae* is associated with pneumonia, abscessation, septicemia, neoplasia, and pyometra, and may facilitate co-infection by other opportunistic bacteria [17,19–21]. Opportunistic infections are significant contributors to southern sea otter mortality [14] and *S*. *phocae* prevalence was reportedly 40.5% within a study of minimally decomposed sea otters necropsied between 2004 and 2010 [18].

Important questions are whether NPMs promote invasion of *S*. *phocae* and other potential bacterial pathogens by damaging the integumentary barrier and if NPMs serve as vectors for transmission of *S*. *phocae* and other bacterial pathogens as they migrate between upper and lower portions of the respiratory tract and invade new hosts. The goals of this study were to (1) assess whether NPM harbor pathogens of veterinary concern and (2) determine the prevalence of *S*. *phocae* among NPM infesting southern sea otters and California sea lions. To explore whether there was evidence of host pathology associated with the pathogens detected in NPM, we present relevant postmortem results from in-depth necropsies conducted as part of a longitudinal study of sea otter mortality [14].

## Materials and methods

### Mite collection

At necropsy each sea otter was classified according to the stranding date, stranding location, sex, age class; pup (<6 months), immature (6 months to <1 year), subadult (1–3 years), adult (4–10 years), and aged adult (>10 year), and nutritional condition (emaciated, poor, fair, good, excellent) [22,23]. NPMs were collected from 16 necropsied southern sea otters and 9 California sea lions from 2007 through 2017 using necropsy methods and criteria described in detail elsewhere [2,14,24]. Briefly, NPMs were gently scooped into cryovials when observed during gross necropsy of fresh or moderately decomposed carcasses. Standardized southern sea otter necropsy protocols included gross examination of the nares, rostral nasal cavity, nasopharynx, oropharynx, soft palate, larynx, trachea, major bronchi, and lungs, and microscopic examination of formalin-fixed tissues from the soft palate, tonsils, oropharynx, and lungs. Additional samples were collected for histopathology if indicated by the presence of moderate to severe gross pathology during necropsy. Although it is beyond the scope of the current study to list the full range of tissue samples that are routinely collected for microscopic examination as part of detailed southern sea otter necropsy, that information has been previously reported [22].

The level of sampling for each case was affected by the extent of postmortem decomposition and other factors, often with reduced sampling for more autolyzed and/or scavenged carcasses, previously frozen animals, or otters infected with the zoonotic fungal pathogen *Coccidioides immitis*. As a result, not all necropsies were comprehensive and therefore neither was NPM collection. Aerobic and/or anaerobic bacterial culture from the nares, nasopharynx, oropharynx, trachea, bronchi, lungs, and other tissues was performed as needed to determine the cause of death, assess infection severity and trace pathogen invasion from wounds or other sources within the body. Bacterial isolation and characterization were performed at the University of California Veterinary Medical Teaching Hospital as previously described [25].

Case selection for bacterial culture was moderated by the presence/absence and relative severity of pathology at gross necropsy and other factors such as the extent of postmortem decomposition, and presence of peri- or postmortem artifact that could interfere with results of bacterial culture or provide spurious results (e.g., perimortem regurgitation, seawater aspiration, or perimortem administration of broad-spectrum antibiotics as part of clinical care). Also considered was the day of necropsy; because bacterial swabs were shipped to a microbiology laboratory bacterial culture and identification, fewer cultures were performed for animals where more than 48 hours of delay were anticipated between sample collection and inoculation onto bacterial culture media. For live-stranded sea otters with NPM infestations, rhinoscopy was performed during hospitalization only when deemed necessary as part of clinical care. Live-stranded otters were only included in this study if ultimately, they died and were submitted for necropsy.

Because NPM were collected opportunistically from marine mammals that died of natural causes or were euthanized by a veterinarian for clinical reasons, this study was verbally exempt from review by the Institutional Animal Care and Use Committee at the University of California Davis. NPM samples were either immediately preserved in 70% ethanol or stored at −20°C prior to placement in 70% ethanol. Individual mites were removed from storage vials, manually separated from any residual host tissue, rinsed with 70% ethanol, and then stored in 70% ethanol. Mites were examined using light microscopy on a stereo zoom microscope (Laxco, Mill Creek, WA) to identify stage- and species-specific anatomic features based on published morphological criteria [3,5,8,26,27]. Microscopic examination of tissue sections from formalin-fixed sea otter tissues was performed on an Olympus BX41 compound microscope and microscopic lesions were photographed using an Olympus DP73 digital camera (Olympus, San Jose, CA).

### DNA extraction

Individual larval or adult NPMs were placed in sterile microcentrifuge tubes and incubated briefly at 56˚C until all residual ethanol had evaporated. Sterile needles were used to pierce each NPM to aid in buffer penetration during tissue lysis. DNA extraction was performed on single mites using the QIAamp DNA Micro Kit (Qiagen, Valencia, CA) under the protocol for tissue and eluted with 40μL Buffer AE. As described below, DNA from NPMs was either subjected to nested conventional PCR detection of *S*. *phocae* or 16S profiling, but not both.

### Conventional PCR

Nested conventional polymerase chain reaction (nPCR) of the 16S ribosomal RNA (16S rRNA) gene was amplified using GoTaq Green Master Mix (2X, Promega, Madison, WI) with primers and cycling conditions as previously described [28]. PCR products were visualized on a 1.5% agarose gel stained with Gel Red (Biotium, Hayward, CA). A synthesized gBlock Gene Fragment (Integrated DNA Technologies, San Diego, CA) containing a 927 bp sequence of the 16S rRNA gene of *S*. *phocae* from GenBank accession AF235052 was used as a positive control. Molecular biology grade nuclease-free water served as a no-template control. Samples with a band of 900 bp were considered positive. The specificity of the primer set used in this investigation (PX1, PX2) for *S*. *phocae* as opposed to other beta-hemolytic Streptococcci was previously evaluated using five reference strains of *S*. *phocae* as positive controls, and multiple strains of each of the species *S*. *pyogenes*, S. *agalactiae*, *S*. *equi subsp*. *equi*, *S*. *equi subsp*. *zooepidemicus*, *S*. *dysgalactiae subsp*. *equisimilis* (serogroup G), *S*. *dysgalactiae subsp*. *dysgalactiae* (serogroup C), *S*. *dysgalactiae subsp*. *dysgalactiae* (serogroup L), *S*. *canis*, S. uberis, *S*. *parauberis*, *S*. *porcinus*, *S*. *suis*, *S*. *iniae*, *S*. *difficilis*, and *Lactococcus garvieae* as negative controls [29].

### 16S profiling by 16S rRNA massively parallel amplicon sequencing

Among the 16 sea otters sampled in this study, 4 underwent detailed necropsy (Table 1) as part of a longitudinal study on sea otter mortality [14]. All cases had chronic moderate to severe nasopulmonary acariasis where larval and adult NPM were collected simultaneously, enabling comparison of the NPM bacterial community across life stages. As part of routine necropsy, subsamples of all major tissues were also collected for histopathology as previously described [22]. In some cases, additional diagnostic tests were performed, including bacterial culture, assessment of postmortem tissue or body fluids for the biotoxins domoic acid and microcystin, and screening of postmortem serum for antibodies to the apicomplexan protozoa *Toxoplasma gondii* and *Sarcocystis neurona*, as previously described [14]. Following completion of histopathology and diagnostic testing, the primary and any contributing cause(s) of death were determined for all 4 sea otters as described [14]. The relative significance of nasopulmonary acariasis relative to all other necropsy findings was noted for each case (Table 1).

**Table 1:** Perimortem clinical data and results of necropsy and histopathology for 4 radio-tagged southern sea otters (*Enhydra lutris nereis*) with nasopulmonary mite infestations that stranded from 2014 through 2016.

Sea otter number	4349–04	7395–15	5229–08	7139–14
Age	Aged adult	Aged adult	Aged adult	Adult
Sex	Female	Male	Female	Female
Perimortem clinical history	Euthanized at admission, no antibiotic therapy	Euthanized at admission, no antibiotic therapy	Found fresh dead, no antibiotic therapy	Died within 24 h, one dose of intramuscular penicillin G total
Bacterial culture at necropsy	None	None	Transmitter pocket & pericardial fluid (Aerobic)	Right retropharyngeal & inguinal lymph nodes (Aerobic/anaerobic)
Culture results	N/A	N/A	Transmitter: Small #s *Vibrio* sp., pericardial fluid: No growth	Lymph nodes: Small #s *Vibrio* sp.& *E*. *coli* (no anaerobes)
Primary COD	Cardiomyopathy	Cardiomyopathy	Mating trauma	Possible microcystin intoxication
Primary sequelae	Heart failure	Heart failure	Bacterial pneumonia, septicemia	Coagulopathy & possible hepatic encephalopathy
Secondary COD	Systemic sarcocystosis and/or toxoplasmosis	Severe dental disease	End lactation syndrome	Cardiomyopathy
Secondary sequelae	None	None	None	Heart failure
Tertiary COD	End lactation syndrome	Emaciation	Cerebral larva migrans	End lactation syndrome
Tertiary sequelae	None	None	None	None
Quaternary COD	**Nasopulmonary acariasis**	**Nasopulmonary acariasis**	Domoic acid intoxication	**Nasopulmonary acariasis**
Quaternary sequelae	Regional lymphadenitis and bacterial spread	Regional lymphadenitis and bacterial spread	None	Regional lymphadenitis and bacterial spread
Quinary COD	Cerebrum: Possible oligodendroglioma	Gastric ulcers/erosions and melena	**Nasopulmonary acariasis**	Domoic acid intoxication
Quinary sequelae	None	None	Regional lymphadenitis and bacterial spread	None
Comments	Both retropharyngeal & axillary LNs reactive on histopathology. The axillary LN contains small clumps of bacterial cocci.	Retropharyngeal LN reactive. Axillary LN not examined microscopically.	Retropharyngeal LN reactive.	Both retropharyngeal & axillary LNs reactive. Right retropharyngeal LN culture-positive for *Vibrio* spp. and *E*. *coli*.

COD = cause of death, LN = lymph node.

Genomic DNA of 69 NPM collected from these 4 necropsied southern sea otters was normalized to a concentration of 9-12ng/μL and stored at -20C prior to amplification and sequencing for bacteriome characterization. DNA from up to 11 larvae or adult mites (Table 2) were pooled for each otter and given a unique barcode label. DNA from larvae and adults was barcoded separately, for a total of 8 pools, to enable characterization of bacterial community composition in relation to mite life stage. Sequences homologous to bacterial 16S rRNA were assembled into contigs. Library preparation was done using the Ion 16S™ metagenomics kit (A26216, ThermoFisher Scientific, Waltham, MA) following manufacturer’s instructions. Briefly, amplicons were made from six 16S rRNA hypervariable regions (V2, V3, V4, V67, V8, V9), then adapters and bar-codes were added. The library concentration was determined by Bioanalyzer (5067–4626, Agilent Technologies, Santa Clara, CA). Barcoded DNA from larval and adult mites from each sea otter was pooled for emulsion PCR at concentrations of 100nM each, allowing for a single sequencing workflow. Clonally amplified DNA was templated into Ion Sphere™ Particles (ISPs) using the Ion Personal Genome Machine (PGM)™ Hi-Q™ View OT2 Kit (A30044, ThermoFisher) on the Ion OneTouch™ 2 instrument, following manufacturer’s instructions. ISPs were enriched on the Ion OneTouch™ ES following manufacturer’s instructions. Sequencing was carried out on the Ion PGM™ using the Ion PGM™ Hi-Q™ View Sequencing kit with the Ion 314™ Chip v2 following manufacturer’s instructions. Briefly, the PGM™ went through initialization to achieve correct pH in the different wash solutions and deoxyribonucleotide triphosphates (dNTP’s). The Ion 314™ Chip v2 was loaded with the ISP’s and the run started according to the manufacturer’s directions. Generated sequences were analyzed using the Ion 16S™ metagenomics analyses module within the Ion Reporter™ software that enables a rapid and semi-quantitative assessment of bacterial samples.

**Table 2:** Number of nasopulmonary mites, mapped reads, and operational taxonomic units (OTUs) of bacteria generated by 16S rRNA massively parallel amplicon sequencing of mites infesting four southern sea otters (*Enhydra lutris nereis*) necropsied during 2014 and 2015 in California.

Sea otter number	Number of mites (juvenile/adult)	Number of mapped reads (juvenile/adult)	Number of OTUs^a^ (juvenile/adult)	Month and year of stranding	Stranding location within Monterey Bay, California
4349–04	20 (10/10)	31,954 (14,057 / 17,897)	17 (9/17)	May 2015	Moss Landing
7395–15	17 (6/11)	41,029 (18,590 / 22,439)	29 (23/24)	March 2015	Moss Landing
5229–08	20 (10/10)	27,121 (12,568 / 14,553)	89 (78/60)	May 2015	Monterey Harbor
7139–14	12 (2/10)	167,647 (22,128 / 145,519)	203 (87/202)	May 2014	Monterey Harbor
**Total**	**69 (28/41)**		**205 (197/303)**		

^a^ Parentheses may include duplicate OTUs found in both pools.

Taxonomical assignments of Operational Taxonomic Units (OTUs) were determined by consensus from all six hypervariable 16S rRNA regions. Multiple taxonomic assignments (slash calls) were reduced to the next highest taxonomic level and condensed into a single OTU (e.g., all *Vibrio* “slash calls” became *Vibrio* sp. and were synonymized with all other genus-only *Vibrio* calls). Descriptive statistics and measures of alpha diversity were calculated using the package phyloseq in R software for statistical computing [30].

The frequency of occurrence for each bacterial taxon was calculated as the number of pools in which the taxon was present divided by the total number of pools.

Differences in count data of mapped reads and OTUs among juvenile and adult pools were assessed using a t-test; and among sea otters using ANOVA. All statistical analyses were performed in R software for statistical computing, with p-values <0.05 considered significant.

## Results

### Mite identification

A total of 250 NPMs from 25 marine mammals were examined in this study. The 204 NPMs obtained from 16 southern sea otters were identified as *H*. *halichoeri*, whereas the 46 NPMs from 9 California sea lions were all *O*. *attenuata* (Table 3).

**Table 3:** Detection of *Streptococcus phocae* bacteria via conventional PCR in nasopulmonary mites collected during marine mammal necropsy in California from 2007 through 2017.

Host species	Total hosts	% hosts with PCR-positive mites (95% CI)	Mite species	Range of mites per host	Total mites	% PCR-positive mites (95% CI)
*Enhydra lutris nereis*	16	87.5% (60.4–97.8%)	*Halarachne halichoeri*	1–29	135	43% (34.6–51.8%)
*Zalophus californianus*	9	66.7% (30.9–91%)	*Orthohalarachne attenuata*	3–11	46	19.6% (9.8–34.4%)
Total	25	80% (58.7–92.4%)			181	37% (30.1–44.5%)

### *Streptococcus phocae* detection using nPCR

A total of 181 NPMs from 25 marine mammals (Table 3) were subjected to nPCR for *S*. *phocae*. Of these, 58/135 NPMs (43%) from 14/16 otters (87.5%) and 9/46 NPM (19.6%) from 6/9 sea lions (66.7%) were PCR-positive for *S*. *phocae* (Table 3). The overall prevalence of *S*. *phocae* detection across all 181 NPM was 37%.

### Bacteriome description based on 16S profiling

The number of readable sequences from the 8 pools of NPM ranged from 12,568 to 145,519. A total of 267,751 mapped reads (Table 2) were initially assigned to 544 bacterial taxa. Raw Ion Reporter™ results are provided in S1 File. After accounting for ambiguous or duplicate assignments, 205 unique bacterial taxa remained (Table 2). A full list of unique bacterial taxa can be found in S2 File. The 10 most abundant taxa were primarily organisms from the families Mycoplasmataceae (especially *Mycoplasma phocidae*), and Vibrionaceae (especially *Vibrio cyclitrophicus* and *Photobacterium damselae*), but also included *Staphylococcus schleiferi*, *Pasteurella multocida*, and *Propionibacterium acnes* (Table 4). Together, these 10 taxa represented 80.7% of all mapped reads. Organisms from the family Mycoplasmataceae occurred most frequently and accounted for 41% of all mapped reads. Organisms from Vibrionaceae accounted for 22.7% of all mapped reads. Other taxa found in at least 5 of the 8 pools included Pasteurellaceae, Vibrionaceae, Staphylococcaceae, Flavobacteriaceae, Burkholderiaceae, Fusobacteriaceae, Moraxellaceae, and Propionibacteriaceae (Table 5). In contrast, 98 taxa representing 58 different families were unique to NPMs from a single otter identified as SO7139-14.

**Table 4:** Ten most abundant operational taxonomic units (OTUs) of bacteria detected by 16S rRNA massively parallel amplicon sequencing in pools of nasopulmonary mites infesting 4 southern sea otters (*Enhydra lutris nereis*) necropsied during 2014 and 2015 in California.

OTU name	Type species	% of mapped reads
Mycoplasmataceae	*Mycoplasma* spp.	19.0%
Mycoplasmataceae		15.1%
Vibrionaceae	*Vibrio* spp.	14.5%
Staphylococcaceae	*Staphylococcus schleiferi*	7.7%
Pasteurellaceae	*Pasteurella multocida*	7.2%
Mycoplasmataceae	*Mycoplasma phocidae*	6.9%
Vibrionaceae		3.8%
Vibrionaceae	*Photobacterium damselae*	2.4%
Propionibacteriaceae	*Propionibacterium acnes*	2.3%
Vibrionaceae	*Vibrio cyclitrophicus*	2.0%

Many bacteria in NPMs could not be identified beyond the genus or family level.

**Table 5:** Most frequent operational taxonomic units (OTUs) of bacteria detected by 16S rRNA massively parallel amplicon sequencing across 8 pools of nasopulmonary mites infesting 4 southern sea otters (*Enhydra lutris nereis*) necropsied during 2014 and 2015 in California.

OTU name	Frequency of occurrence
Mycoplasmataceae	100%
*Mycoplasma* spp.	100%
*Mycoplasma phocidae*	87.5%
*Pasteurella multocida*	87.5%
Vibrionaceae	75%
*Staphylococcus schleiferi*	75%
Flavobacteriaceae	62.5%
*Burkholderia* spp.	62.5%
*Fusobacterium* spp.	62.5%
*Moraxella* spp.	62.5%
*Pasteurella* spp.	62.5%
*Propionibacterium acnes*	62.5%
*Psychrobacter aestuarii*	62.5%
*Psychrobacter* spp.	62.5%

Frequency of occurrence was calculated as the number of pools in which the taxon was present, divided by the total number of pools and expressed as a percentage.

**Fig 1:**
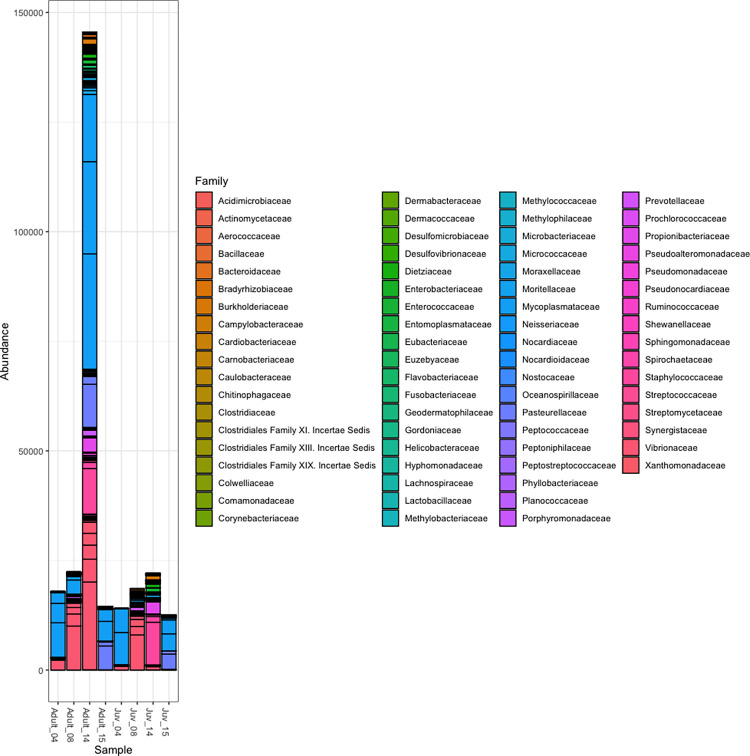
Plot of operational taxonomic units (OTUs) of bacteria from 8 pools of nasopulmonary mites infesting 4 southern sea otters (*Enhydra lutris nereis*) by taxonomic family.

There were consistently more mapped reads from pools of adult NPMs than juvenile NPMs, though this trend was not statistically significant (P = 0.338). Similarly, although the pool of adult NPMs from otter SO7139-14 had considerably more bacterial taxa than any other pool (Fig 1), neither mapped reads nor OTU count differed significantly by NPM life stage (P = 0.596) or by otter (P = 0.419).

A total of 16 organisms with known or suspected pathogenic potential were identified among the 205 unique OTUs (Table 6), many of which impact the respiratory system of marine mammals and some that can infect humans. Notably, *S*. *phocae* was detected in both the juvenile and adult pools of NPM from otter SO7139-14.

**Table 6:** Operational taxonomic units (OTUs) of bacteria with suspected or confirmed pathogenic potential detected in nasopulmonary mites from southern sea otters (*Enhydra lutris nereis*) necropsied in California during 2014 and 2015.

Phylum	OTU	Hosts	Isolation location	Associated pathology	Reference
Actinobacteria	*Arcanobacterium phocae*	Sea otters, seals, sea lions, elephant seals, dolphins	**Respiratory system**, systemic	Pneumonia, septicemia, inflammation, abscess, **fatal lesions in sea otters**	[31,32]
Bacteroidetes	*Ornithobacterium rhinotracheale*	Poultry, wild birds	**Respiratory system**	Airsacculitis, pneumonia	[33,34]
Firmicutes	*Clostridium perfringens*	Sea otters, marine invertebrates	Digestive system	Necrotizing enteritis, **fatal lesions in sea otters**	[14,25,35]
	*Helcococcus ovis*	Livestock, horses	**Respiratory system,** circulatory system	Pneumonia, inflammation	[36–39]
	*Staphylococcus schleiferi*	Sea otters, seals, penguins, dogs, humans, wild birds	Systemic	Inflammation, abscess, **fatal lesions in sea otters**	[14,40]
	*Streptococcus dysgalactiae*	Whales, fish, livestock, humans	Systemic	Septicemia, necrotic ulcers, inflammation, abscess	[41,42]
	*Streptococcus phocae*	Sea otters, Steller sea lions and other pinnipeds, salmonids, mink,	**Respiratory system**, systemic	Pneumonia, septicemia, neoplasia, pyometra, **fatal lesions in sea otters**	[14,18,43–49]
Fusobacteria	*Fusobacterium mortiferum*	Humans	**Respiratory system**, digestive system	Abscess, septicemia	[50,51]
	*Fusobacterium necrophorum*	Sea otters, livestock, antelope, marsupials, humans	**Respiratory system**	Necrobacillosis, pneumonia, **fatal lesions in sea otters**	[14,50–57]
Proteobacteria	*Helicobacter* sp.	Sea otters, seals, sea lions, fur seals, dolphins, wild birds	Digestive system	Ulcers	[58–63]
	*Campylobacter* sp.	Sea otters, seals, elephant seals	Digestive system	Inflammation, **fatal lesions in sea otters**	[14,25,64,65]
	*Mannheimia varigena*	Cattle, sheep, swine, leeches, coral	**Respiratory system**, digestive system	Inflammation	[66–68]
	*Pasteurella multocida*	Sea otters, seals, sea lions, walrus, livestock, dogs, cats, poultry and wild birds, rabbits, chimpanzees, komodo dragons	**Respiratory system**	Pneumonia, septicemia, inflammation, **fatal lesions in sea otters**	[14,40,69–73]
	*Photobacterium damselae*	Crustaceans, mollusks, cetaceans, humans, sharks, seafood	Systemic	Bacteremia, septicemia, necrotizing fasciitis	[74,75]
	*Vibrio parahaemolyticus*	Sea otters, dolphins, shrimp, fish, humans	Systemic, digestive system	Cholera, **fatal lesions in sea otters**	[14,25,76–81]
Tenericutes	*Mycoplasma phocidae*	Seals	**Respiratory system**, systemic	Inflammation, ulcers	[82–85]

### Sea otter pathology

Findings from gross necropsy and histopathology for the 4 sea otters where NPM were collected for bacteriome assessment are summarized in Table 1. In all cases, chronic NPM infestation was considered a contributing cause of death. The regional (draining) lymph nodes were enlarged and chronically inflamed, and bacterial spread to a regional lymph node was confirmed microscopically in one case (Table 1). All three female sea otters in the sample also had mating-associated facial trauma, including nose wounds that could contribute to observed respiratory and lymph node pathology and bacterial spread.

**Fig 2:**
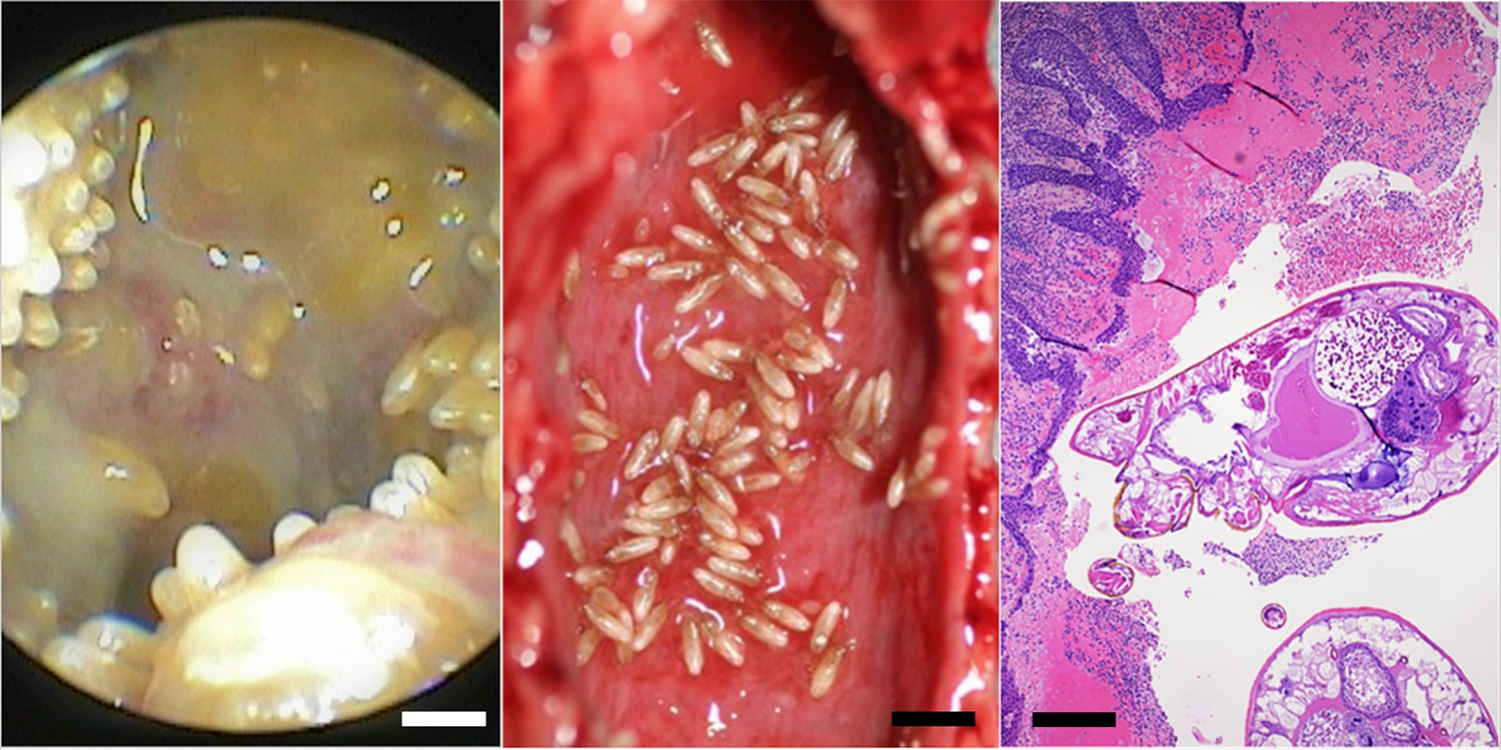
Examples of pathology associated with nasopulmonary mite infestations in southern sea otters (*Enhydra lutris nereis*). (A) Example rhinoscopic view of the nasopharynx from a live southern sea otter infested with nasopulmonary mites, showing abundant mucopurulent and variably hemorrhagic fluid surrounding adult nasopulmonary mites covering the ventral and lateral nasopharyngeal mucosa (Bar = 0.75 mm); (B) Diffusely inflamed, congested and mildly edematous nasopharyngeal mucosa in a necropsied sea otter with severe nasopulmonary mite infestation (Bar = 4 mm); (C) Example microscopic view of adult nasopulmonary mites attached to an inflamed, edematous and hemorrhagic nasopharyngeal mucosa (Hematoxylin and eosin stain, Bar = 250 μm).

In all cases, larval mites were most numerous in the rostral nasal cavity at gross necropsy, while adults were common in the nasopharynx, oropharynx, larynx, and trachea, where they remained attached to the mucosa. In some cases, abundant mucopurulent and sparsely hemorrhagic fluid surrounded the attached adult mites in the nasopharynx (Fig 2A); similar fluid was sometimes present in the nasal cavity, trachea, and bronchi. The nasopharyngeal mucosa was diffusely red and mildly edematous. Attached adults sometimes formed a continuous mass of mites covering the ventral and lateral nasopharyngeal mucosa (Fig 2A and 2B), with additional mites in the trachea and bronchi. The regional lymph nodes, especially the retropharyngeal and axillary lymph nodes, were often moderately to markedly enlarged, solid on palpation, and tan or tan-red-mottled. Sea otters with chronic, severe mite infestations often had one or more small (2–4 mm diameter), well-circumscribed, flat or mildly depressed white spots scattered throughout the pulmonary pleura.

**Fig 3:**
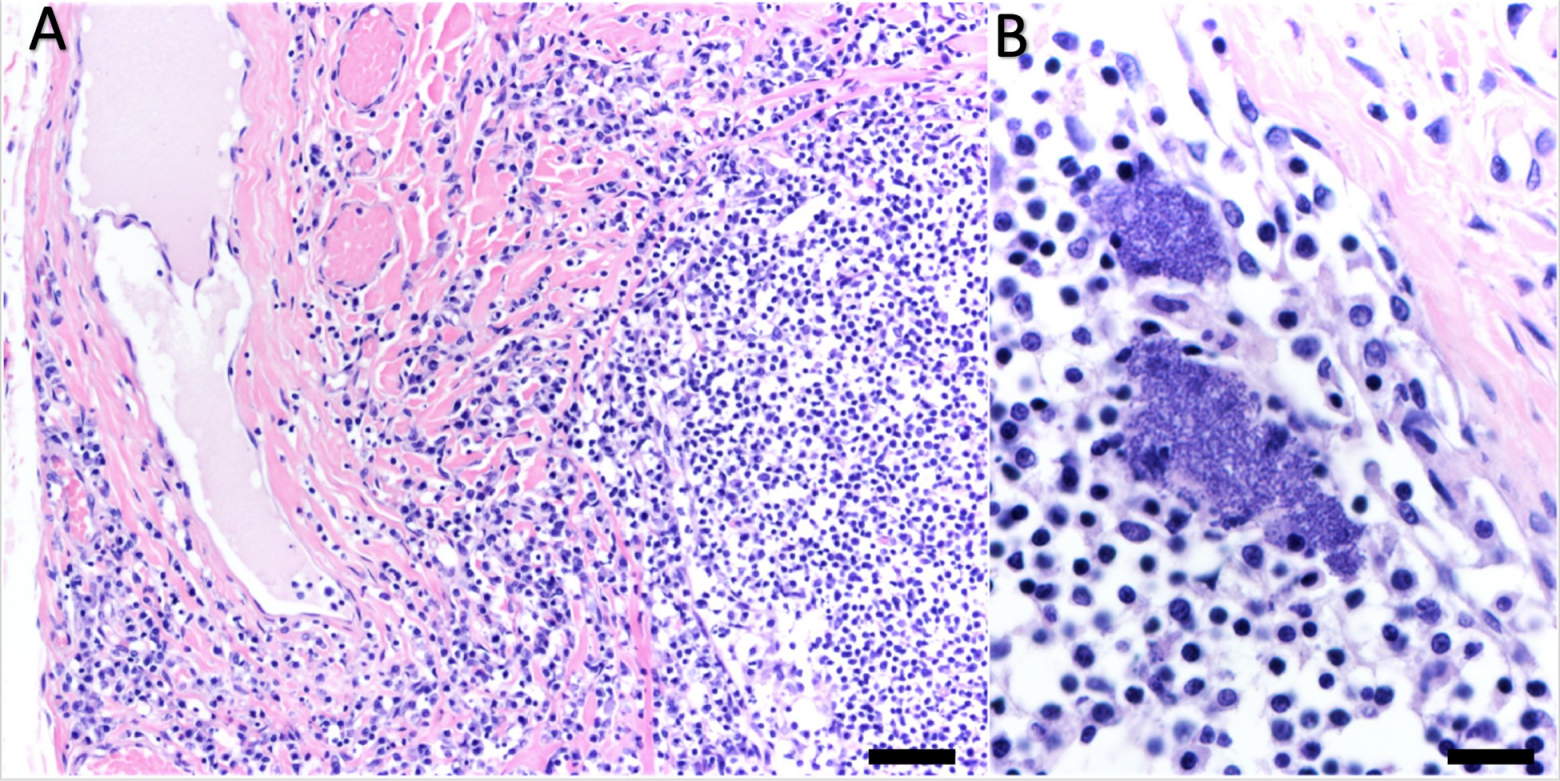
Microscopic views of perinasal draining lymph nodes from southern sea otters (*Enhydra lutris nereis*) with nasopulmonary mite infestations. (A) Chronically inflamed retropharyngeal lymph node with lymphatic dilation (top left) and marked expansion of the lymph node capsule by a mixed inflammatory infiltrate (left). (B) Inflamed axillary lymph node containing two dense clusters of bacterial cocci (putative streptococci) in the superficial cortex. Both sections hematoxylin and eosin stain, Bar = 100 μm (A) and 40 μm (B).

On histopathology the nasopharyngeal mucosa was often diffusely inflamed, congested and mildly edematous (Fig 2C). The inflammatory infiltrate was composed of neutrophils, plasma cells, lymphocytes, macrophages, and sparse eosinophils, often accompanied by patchy submucosal hemorrhage. Transmigrating neutrophils were common in the mucosa, and numerous neutrophils were admixed with proteinaceous fluid and hemorrhage in the nasopharyngeal lumen. The dorsal soft palate in sea otters is lined by a thin layer of pseudostratified columnar epithelium, sometimes with small patches of ciliated pseudostratified columnar epithelium, depending on the sample location. Where adult mites were densely packed along the dorsal soft palate, the pseudostratified columnar epithelium exhibited an irregular, shaggy appearance with sparse surface erosion and patchy squamous metaplasia. The regional lymph nodes, especially the retropharyngeal and axillary lymph nodes exhibited chronic lymphadenitis, characterized by mild to moderate numbers of neutrophils, plasma cells, lymphocytes and macrophages within the capsule, trabeculae, lymphatics and subcapsular and medullary sinuses (Fig 3A). The lymph node capsule was often mildly thickened and fibrotic. This chronic inflammation was associated with moderate diffuse paracortical lymphoid hyperplasia (Fig 3A). The tonsils were often enlarged and inflamed, with moderate lymphoid hyperplasia.

Bacterial culture was performed at necropsy for one of the four sea otters (Table 1); in this case the retropharyngeal lymph node was culture-positive for *Vibrio* spp. and *E*. *coli*. The axillary lymph node of another otter contained small clumps of bacterial cocci arranged in chains (putative streptococci) on histopathology (Fig 3B).

## Discussion

NPM are common respiratory parasites of marine mammals that cause pulmonary irritation and impairment [10–13]. Although NPM often co-occur with bacterial infection [12,13,16], their role in the transmission of bacteria had not been explored. In this study, NPM were collected from both southern sea otters and California sea lions during necropsy to determine whether they harbored bacterial organisms with known pathogenic potential in marine mammals. PCR was used to directly detect *S*. *phocae* in NPM from both sea otters and sea lions as a specific pathogen of interest whereas 16S profiling was used to investigate the broader bacteriome of pools of larval or adult NPM from a subset of sea otters. This subset of otters was chosen because sufficient numbers of both NPM life stages were collected simultaneously, allowing for comparison of bacterial community between life stages.

More than one third of NPMs sampled were positive for *S*. *phocae* using nPCR, and most marine mammal hosts harbored mites that were *S*. *phocae* positive. The primers used in the current study were previously demonstrated as effective at distinguishing *S*. *phocae* from other closely related beta-hemolytic Streptococci [29]. For one otter, *S*. *phocae* was successfully detected in *H*. *halichoeri* using both 16S profiling and nPCR.

Beta-hemolytic streptococci, including *S*. *phocae* are some of the most common opportunistic bacterial pathogens of southern sea otters and other marine mammals [14]. These bacteria commonly colonize damaged skin, often resulting in abscessation, bacteremia, or sepsis [18] and have been associated with fatal lesions in otters [35]. Breaks in host integument due to trauma and other causes was significantly associated with risk of infection in an epidemiologic study of *S*. *phocae* in sea otters [18]. Internal lesions were assessed cumulatively as perforations in the mucosa of the gastrointestinal, respiratory, or urogenital tract. However, specific associations with NPM infestation were not assessed, and NPMs were reported during gross necropsy or in archival photos in only four *S*. *phocae-*positive otters as part of that prior study [18]. An insufficient number of individuals with NPM-associated lesions were included in the prior study to conclude whether mucosal damage from NPMs facilitates infection with *S*. *phocae*. Interestingly, *S*. *phocae* and other beta-hemolytic streptococci are commonly isolated from the nares and nasopharynx at necropsy, including nose wounds of adult female sea otters that develop as a result of copulatory activity (M. Miller, unpub. data). In addition, for one of the four sampled sea otters in the current study (an adult female with a nose wound and severe NPM infestation), a regional lymph node contained small clumps of bacterial cocci arranged in chains (putative streptococci) on histopathology (Fig 3B). Additional studies are needed to clarify the relationship between NPM infestation and the potential for spread of *S*. *phocae* and other beta-hemolytic Streptococci within and between sea otters and other marine mammals.

The bacteriome of the *H*. *halichoeri* sampled in this study was dominated by organisms from the family Mycoplasmataceae, a family consisting of *Mycoplasma* and *Ureaplasma* genera. These small, pleomorphic bacteria lack a cell wall [86] and have exceptionally short genomes [87]. Many Mycoplasmataceae are pathogenic in humans and animals, including *M*. *phocicerebrale* which is associated with zoonotic skin infections (i.e. seal finger) [82]. *Mycoplasma* species, especially *M*. *phocidae*, have been reported in harbor seals and grey seals [83,84] and are commonly associated with infected wounds of the respiratory tract [83–85,88]. *Mycoplasma phocidae* has also been found on the teeth of pinnipeds and in bite wounds [84] suggesting it is an opportunist capable of causing severe infection through breaks in host integument. NPMs are found in these same pinniped species; mechanical transfer of *Mycoplasma* species by mites may be a route for bacterial spread between pinniped hosts, and mites may facilitate tissue infection through damage to the mucosa secondary during feeding. A majority of the Mycoplasmataceae in NPMs could not be identified beyond the genus or family level, though this is not unusual since there is a great diversity of undescribed *Mycoplasma* species, including many that are associated with respiratory disease [84,89]. *Mycoplasma* strains have previously been isolated from the oropharynx of southern sea otters at necropsy including a purportedly novel *Mycoplasma* species, for which the name *Mycoplasma enhydrae* sp. nov. has been proposed [90]. Given that 19% of mapped reads in the current study were *Mycoplasma* spp., it is highly possible that *Mycoplasma enhydrae* sp. nov. was present in NPM.

The second most prolific bacterial group identified in *H*. *halichoeri* were from the family Vibrionaceae. There have been several revisions to taxonomy within Vibrionaceae; the family currently consists of two genera, *Vibrio* and *Photobacterium*, which are motile, gram-negative bacteria that generally require seawater for growth [91]. These bacteria are commonly associated with marine and estuarine environments and are capable of infecting a wide variety of aquatic organisms [91]. Although some Vibrionaceae in NPMs could not be identified beyond the family or genus level, the species *V*. *cyclitrophicus* and *P*. *damselae* were abundant. A prior study of bacterial isolates in southern sea otters identified *P*. *damselae* in lung tissue [74]. Certain subspecies of *P*. *damselae* can cause wound infections in marine animals and hemorrhagic septicemia or severe necrotizing fasciitis in humans [75]; however, subspecies identification of *P*. *damselae* from NPMs was not possible for the current study. The fecal pathogen *V*. *parahaemolyticus* was also detected in NPMs but was not abundant. This pathogen can cause severe gastroenteritis in humans [76], and is increasingly reported among sick or stranded marine mammals [77], including sea otters [25]. A sea otter with *Vibrio* spp.-positive NPM in the current study (SO7139-14) was also culture-positive for *Vibrio* spp. in a regional lymph node (Table 1). Fatal lesions associated with *Vibrio* spp., including *V*. *parahaemolyticus*, have been documented in southern sea otters [14].

Numerous organisms with pathogenic potential were detected in *H*. *halichoeri*, including several associated with disease in sea otters and pinnipeds (Table 6). Most of these organisms are considered opportunistic pathogens that rely on breaks in host integument or mucosa, in some cases causing severe systemic disease or death. In a recent long-term study of sea otter mortality and morbidity, bacterial invasion was a primary or contributing cause of death for 12% of southern sea otters and overall 68% of otters had bacterial infection as a cause of death or sequela [14]. Seven of the pathogens detected in NPM were found to be associated with fatal lesions in that study: *S*. *phocae*, *P*. *multocida*, *Campylobacter* spp., *V*. *parahaemolyticus*, *Fusobacterium necrophorum*, and *Clostridium perfringens* [14]. Some of the organisms detected in NPM also represent biological pollution of microbes from land to sea via coastal runoff [35]. Additionally, to our knowledge this is the first report of *F*. *mortiferum*, *Helcococcus ovis*, and *Ornithobacterium rhinotracheale* in the marine environment.

Numerous bacteria harbored by *H*. *halichoeri* have also been identified in other mites. *Staphylococcus* species have been reported in the red poultry mite (*Dermanyssus gallinae)*, the mold or cheese mite (*Tyrophagus putrescentiae)*, and the chigger *Leptotrombidium imphalum* [92–95]. Though there were several *Staphyloccocus* species found in *H*. *halichoeri*, the most common was *S*. *schleiferi* which is most often associated with humans and dogs but has also been reported in pinnipeds as well as numerous other animals [40]. This bacterium is thought to be primarily commensal, but some subspecies are opportunistic pathogens of humans and dogs. In sea otters, *S*. *schleiferi* has been associated with fatal lesions [14]. *Pasturella multocida* represented almost 7% of all mapped reads in NPM and has also been found in red poultry mites. *Pasturella multocida* is an economically important pathogen that causes fowl cholera in birds and pneumonia or hemorrhagic septicemia in a diversity of mammalian hosts [96] including fatal lesions in sea otters [14]. This bacterium often invades the lower respiratory tract and can spread systemically [97]. *Propionibacterium acnes*, a human skin commensal that can cause acne as well as other infections [98], is another abundant bacterium in NPMs that has also been detected in *L*. *imphalum*. The presence of *P*. *acnes* may represent a human contaminant in NPM or a transient, yet relevant, passenger. Interestingly, no Rickettsiales were detected in NPMs despite this bacterial order being commonly associated with terrestrial Acari [92,93,95,99,100].

Interpretations of the composition of microbial communities can vary based on the method of amplicon generation and the choice of bioinformatic pipeline used for analysis [101,102]. Characterization of dominant microbiota in the house dust mite (*Dermatophagoides farina*) microbiome varied among studies employing different approaches to amplicon generation and within a study that compared results across three different bioinformatic pipelines [101]. The 16S profiling approach used in this NPM study has been demonstrated as a superior method of characterizing microbiomes, particularly when higher sensitivity for finer resolution is desired [102]. Our findings, based on a limited sample set, suggest that approximately 80% of the NPM bacteriome is comprised of just 10 OTUs (Table 4). Similarly low bacteriome diversity has been reported in red poultry mites [92], house dust mites [101,103], and the chigger *L*. *imphalum* [95].

There are other possible alternative explanations for the microbiota detected in NPMs in this study that are external to NPMs. As endoparasites, NPMs are anchored into host integument so it is possible that the bacterial community reported here represents surface contamination by host microbiota. Care was taken to separate host tissue from NPMs and they were rinsed with ethanol prior to DNA extraction. Ethanol was chosen over a mild sodium hypochlorite (bleach) solution commonly used in preparing ticks for microbiome analysis [104] due to concerns that such a caustic product would damage the soft-bodied halarachnid mite specimens. However, future NPM studies should explore whether repeated washings with phosphate buffered saline [92,95,105] or DNA Away [106] are superior approaches for surface decontamination. Regardless of whether some of the bacterial taxa reported here are from the mite surface, neither the value of these bacteria, nor their association with mites, should immediately be dismissed. The mammalian skin microbiome is important to mammalian immune defense [107,108] so the surface bacteria of parasites may also play an important role in pathogen transmission and spread [109].

A primary challenge for identifying parasites as potential disease vectors is untangling parasite and host microbial communities because parasites imbibe portions of the host’s microbiome during feeding or are surface contaminated when anchored in host tissues. In populations of red poultry mites, Hubert et al. [92] compared microbial communities among eggs, larvae, and engorged nymphs/adults and found *Bartonella* spp. in all life stages. These results suggest that red poultry mites are not only getting *Bartonella* bacteria from the host during feeding, but that potentially maternal (transovarial) transmission is occurring, and the parasite-pathogen relationship may continue through life stages (transstadial transmission). Comparing the bacterial communities of different mite life stages provides more information about the relationships between parasite and associated opportunistic pathogens and clarifies the potential role of parasites in disease transmission.

No significant differences in mapped reads nor OTU count were detected by NPM life stage or by otter, though our sample sizes were low for resolution at this level. Other mite bacteriome studies have detected differences in bacterial community composition between life stages. In a study of red poultry mites, Hubert et al. [92] found that counts of OTUs and bacterial diversity were 2X higher in eggs and larvae compared with adults and nymphs. Sample site also significantly influenced bacterial community composition, which the authors attributed to variations in site-specific farming practices. In a study of red poultry mites, Lima-Barbero et al. [110] reported significant differences in the composition of alphaproteobacterial microbiota by life stage and feeding status. The species composition of bacterial communities of *L*. *imphalum* also differed by life stage [95]. The biology of these mite species differs substantially from that of NPMs. Red poultry mites are hematophagous ectoparasites, but only nymphs and adults blood feed. Similarly, *L*. *imphalum* only blood feeds during the larval stage. Because not all life stages are parasitic, the microbiome of some life stages may be decoupled from that of the host. As obligate endoparasites, NPM consume host lymph during juvenile and adult life stages, making it difficult to distinguish between mite-specific bacteria (e.g., endosymbionts) and bacteria associated with the meal, host, or environment. Additionally, although some mites can be reared in laboratory colonies to investigate possible endosymbiotic microbes and evaluate or manipulate changes in microbial community composition [94,95,101,111], methods for *in vitro* propagation of NPMs have not yet been developed.

Although the bacterial community of *H*. *halichoeri* was largely consistent across all pools, one sample of adult mites from otter SO 7139–14 had markedly higher diversity. In contrast, the bacteriome of the pool of juvenile mites from this same otter more closely resembled that of mites from other otters. It is unclear why the bacteriome of the adult mite pool would differ from the juvenile mite pool from the same host, but it may be related to mite ontogeny. Since adults are sessile, it is possible the mite bacteriome more closely resembles the host bacteriome after longer periods of infestation.

Otter SO7139-14 stranded in poor condition and was hospitalized but died within a day of stranding. During necropsy, mild infestations of both juvenile and adult mites were noted in the oropharynx and nasopharynx; these mites were sampled for evaluation in this study. *Streptococcus phocae* was detected in mites from otter SO7139-14 on both nPCR and 16S profiling, along with several other species of *Streptococcus*, *Vibrio*, *Mycoplasma*, and every organism with pathogenic potential listed in Table 6. Lymph node cultures did not detect any aerobic bacteria, which would include *S*. *phocae*, but did detect very low levels of *Vibrio* spp. and *Escherichia coli*. No bacterial cocci were observed on histopathology. Notably, antibiotics were administered as part of perimortem therapy (Table 1), which could impact the results of bacterial culture and histopathology. Microcystin intoxication was suspected but was not confirmed. *Streptococcus phocae* infection was not detected on culture for any other otters in this study where mites were positive, but very limited bacterial culture was performed. Although several other *S*. *phocae*-positive otters were infested with NPM, no mites were available from these cases for genomic (or nPCR) testing. It is possible that chronic NPM infestation contributed to the bacterial pneumonia and septicemia for sea otter SO5229-08, along with concurrent severe mating trauma (Table 1). Culture was not performed on the six California sea lions harboring *S*. *phocae-*positive NPM from this study, however three had a diagnosis of pneumonia in their perimortem medical notes.

NPM larvae are highly motile and hardy outside of the host [2,6] making them potential vectors for bacterial transmission between hosts [6]. Several mite species have been identified as biological or mechanical vectors of bacterial pathogens. Examples of biological vectors (where bacteria multiply within the mite host), are the mouse mite (*Liponyssoides sanguineus*) which transmits *Rickettsia akari* causing human rickettsialpox [112], and larval mites (chiggers) of the genus *Leptotrombidium*, which transmit *Orientia tsutsugamushi*, causing scrub typhus [95]. Although some other mite species are closely associated with bacterial infections, it is unclear whether they merely create opportunity (i.e. causing physical breaks in host tissues or inhibiting host immune responses) [107,113], mechanically contaminate the site, or serve as hosts for bacterial propagation. Examples include human hair follicle mites (*Demodex folliculorum* and *D*. *brevis*) which are associated with opportunistic infections of *Staphylococcus aureus* and *Streptococcus pyogenes* [114,115], sarcoptic mange mites (*Sarcoptes scabiei*) which cause dysbiosis of the mammalian skin microbiome and facilitates secondary bacterial infections, particularly *Staphylococcus* spp. [107,116], and red poultry mites (*Dermanyssus gallinae*) which are implicated in transmission of several bacterial and viral pathogens [93,117] including transstadial and transovarial transmission of *Salmonella enteritidis* [118]. Sea otters with chronic severe NPM infestation often have mucopurulent and variably bloody fluid surrounding the adult mites in the nasopharynx (Fig 2A), and this fluid can extend into the trachea and bronchi. On histopathology this fluid often contains numerous bacterial cocci and rods. In addition, the mucosal epithelium of heavily parasitized regions is roughened and irregular, with numerous tiny foci of mucosal erosion or ulceration. Collectively our data suggest that NPM may be able to transport opportunistic bacterial pathogens from host to host. In addition, through their feeding activity, these mites provide portals of bacterial entry and spread via direct mucosal damage, mite movement throughout the respiratory tract, and elicitation of mucopurulent fluid containing mites and opportunistic bacterial pathogens that can spread into the lower respiratory tract.

Even if NPMs are not biological vectors of bacterial pathogens, the detection of multiple organisms with confirmed pathogenic potential in or on NPMs appears to confirm their role as potential mechanical vectors. If larvae become contaminated with an opportunistic pathogen in one host, then spread to another host where they open tissue and contaminate the wound, this process could pose significant host health risks. Notably, many mite species demonstrate relatively low host specificity. *Halarachne halichoeri* infestations have been reported in eight marine host species [15,119,120] and *Orthohalarachne attenuata* in fourteen marine host species [15,119], suggesting broad exchange of halarachnid mites among proximate marine mammal populations [2,13,120]. As a result, NPM could mechanically or biologically vector bacteria and other pathogens within and between mammalian host species. NPM infestation could also modify the microhabitat of the upper respiratory tract to favor opportunistic bacterial pathogens.

This study represents several new contributions to the current body of mite microbiome literature. To date most mite microbiome studies have focused on ectoparasitic mites of domestic animals in terrestrial environments. Our study represents the first investigation of the microbial community of mites parasitizing wildlife hosts, of an endoparasitic mite parasitizing any host taxa, and of an acarine in the marine environment adding to a broader understanding of parasite microbiota across contexts. Our results confirm that NPM harbor *S*. *phocae* and other bacterial pathogens of marine mammals. Given that NPMs are globally distributed and relatively abundant across marine mammal populations [12,16,119,120], including imperiled species like the southern sea otter and the Guadalupe fur seal (*Arctocephalus philippii townsendi*) [2,15], the potential for transmission of opportunistic bacterial pathogens is concerning. This potential for pathogen spread between animals should be considered for animal translocation programs, and co-housing of animals in rehabilitation facilities, zoos, aquaria, and oil spill response settings. Because the highly mobile larvae can survive for prolonged periods outside of the host [2,6] and the mites are both directly pathogenic and may spread bacteria between animals, aggressive facility decontamination and periodic animal treatment with acaracides is advised.

Collectively our findings contribute to an improved understanding of the host-parasite microbial community which can inform veterinary treatment, captive animal care, and animal conservation and translocation efforts. Prior captive care is a risk factor for NPM infestation in wild sea otter populations [13], and NPM parasitism can be very high in captivity without regular acaricidal treatment [1,4,121], and NPM are found in marine mammals worldwide [15,119,120]. As a result, our work has important implications for preventing pathogen transmission during captive care, oil spill response, and animal reintroduction efforts globally. Additional studies are needed to investigate the potential environmental and ecological interconnections between *H*. *halichoeri* mites, wild sea otters, sympatric harbor seals (another common host for *H*. *halichoeri*), and the various bacteria encompassed in this study. Because sea otters are a federally listed threatened species that is struggling to achieve population recovery, and animal translocation is being considered as a potential tool to facilitate recovery, this work is strongly merited. Future studies should continue to investigate NPM-host-pathogen relationships to better understand the role these mites play in bacterial infection and the significance of different bacterium in NPM for mite biology.

## Supporting information

S1 File Raw Ion Reporter™ results generated by 16S rRNA massively parallel amplicon sequencing of nasopulmonary mites infesting four southern sea otters (*Enhydra lutris nereis*) necropsied during 2014 and 2015 in California. (XLSX)

S2 File Summary of all operational taxonomic units (OTU) of bacteria generated by 16S rRNA massively parallel amplicon sequencing of nasopulmonary mites infesting four southern sea otters (*Enhydra lutris nereis*) necropsied during 2014 and 2015 in California. (PDF)
