# Supplementary material for: Nasopulmonary mites (Acari: Halarachnidae) as potential vectors of bacterial pathogens, including Streptococcus phocae, in marine mammals
Source: PLoS One. 2022 Jun 16;17(6):e0270009. doi: 10.1371/journal.pone.0270009 (PMC9202935; doi:10.1371/journal.pone.0270009)
Supplement: S2 File — (PDF) [file pone.0270009.s002.pdf]

| ID | Phylum | Class | Order | Family | Genus | Species |
|---|---|---|---|---|---|---|
| OTU1 | Tenericutes | Mollicutes | Mycoplasmatales | Mycoplasmataceae | | |
| OTU2 | Tenericutes | Mollicutes | Entomoplasmatales | Entomoplasmataceae | | |
| OTU3 | Fusobacteria | Fusobacteriia | Fusobacteriales | Fusobacteriaceae | | |
| OTU4 | Proteobacteria | Gammaproteobacteria | Vibrionales | Vibrionaceae | | |
| OTU5 | Firmicutes | Clostridia | Clostridiales | Clostridiales Family XI. Incertae Sedis | | |
| OTU6 | Proteobacteria | Gammaproteobacteria | Cardiobacteriales | Cardiobacteriaceae | | |
| OTU7 | Bacteroidetes | Flavobacteriia | Flavobacteriales | Flavobacteriaceae | | |
| OTU8 | Proteobacteria | Gammaproteobacteria | Pseudomonadales | Moraxellaceae | | |
| OTU9 | Bacteroidetes | Bacteroidia | Bacteroidales | Prevotellaceae | | |
| OTU10 | Firmicutes | Clostridia | Clostridiales | Eubacteriaceae | | |
| OTU11 | Firmicutes | Clostridia | Clostridiales | Lachnospiraceae | | |
| OTU12 | Proteobacteria | Deltaproteobacteria | Desulfovibrionales | Desulfovibrionaceae | | |
| OTU13 | Synergistetes | Synergistia | Synergistales | Synergistaceae | | |
| OTU14 | Firmicutes | Clostridia | Clostridiales | Peptoniphilaceae | | |
| OTU15 | Proteobacteria | Epsilonproteobacteria | Campylobacterales | Helicobacteraceae | | |
| OTU16 | Proteobacteria | Gammaproteobacteria | Pasteurellales | Pasteurellaceae | | |
| OTU17 | Firmicutes | Bacilli | Lactobacillales | Aerococcaceae | | |
| OTU18 | Bacteroidetes | Bacteroidia | Bacteroidales | Porphyromonadaceae | | |
| OTU19 | Firmicutes | Bacilli | Lactobacillales | Enterococcaceae | | |
| OTU20 | Actinobacteria | Actinobacteria | Actinomycetales | Dietziaceae | | |
| OTU21 | Firmicutes | Clostridia | Clostridiales | Peptostreptococcaceae | | |
| OTU22 | Proteobacteria | Gammaproteobacteria | Methylococcales | Methylococcaceae | | |
| OTU23 | Actinobacteria | Actinobacteria | Actinomycetales | Nocardiaceae | | |
| OTU24 | Actinobacteria | Actinobacteria | Actinomycetales | Pseudonocardiaceae | | |
| OTU25 | Proteobacteria | Epsilonproteobacteria | Campylobacterales | Campylobacteraceae | | |
| OTU26 | Actinobacteria | Actinobacteria | Euzebyales | Euzebyaceae | | |
| OTU27 | Cyanobacteria | Nostocophycideae | Nostocales | Nostocaceae | | |
| OTU28 | Firmicutes | Clostridia | Clostridiales | Clostridiales Family XIX. Incertae Sedis | | |
| OTU29 | Firmicutes | Clostridia | Clostridiales | Ruminococcaceae | | |
| OTU30 | Proteobacteria | Alphaproteobacteria | Rhodobacterales | Hyphomonadaceae | | |
| OTU31 | Proteobacteria | Gammaproteobacteria | Alteromonadales | Pseudoalteromonadaceae | | |
| OTU32 | Proteobacteria | Gammaproteobacteria | Alteromonadales | Shewanellaceae | | |
| OTU33 | Proteobacteria | Gammaproteobacteria | Oceanospirillales | Oceanospirillaceae | | |
| OTU34 | Proteobacteria | Alphaproteobacteria | Rhizobiales | Bradyrhizobiaceae | | |

| | | | | | | |
|---|---|---|---|---|---|---|
| OTU35 | Proteobacteria | Betaproteobacteria | Burkholderiales | Burkholderiaceae | | |
| OTU36 | Actinobacteria | Actinobacteria | Actinomycetales | Micrococcaceae | | |
| OTU37 | Proteobacteria | Alphaproteobacteria | Caulobacterales | Caulobacteraceae | | |
| OTU38 | Proteobacteria | Gammaproteobacteria | Enterobacteriales | Enterobacteriaceae | | |
| OTU39 | Proteobacteria | Gammaproteobacteria | Xanthomonadales | Xanthomonadaceae | | |
| OTU40 | Actinobacteria | Actinobacteria | Actinomycetales | Dermacoccaceae | | |
| OTU41 | Actinobacteria | Actinobacteria | Actinomycetales | Microbacteriaceae | | |
| OTU42 | Firmicutes | Bacilli | Lactobacillales | Carnobacteriaceae | | |
| OTU43 | Actinobacteria | Actinobacteria | Actinomycetales | Streptomycetaceae | | |
| OTU44 | Firmicutes | Bacilli | Lactobacillales | Lactobacillaceae | | |
| OTU45 | Proteobacteria | Alphaproteobacteria | Sphingomonadales | Sphingomonadaceae | | |
| OTU46 | Firmicutes | Bacilli | Lactobacillales | Aerococcaceae | Abiotrophia | defectiva |
| OTU47 | Actinobacteria | Actinobacteria | Acidimicrobiales | Acidimicrobiaceae | Aciditerrimonas | |
| OTU48 | Proteobacteria | Gammaproteobacteria | Pseudomonadales | Moraxellaceae | Acinetobacter | |
| OTU49 | Proteobacteria | Gammaproteobacteria | Pasteurellales | Pasteurellaceae | Actinobacillus | minor |
| OTU50 | Proteobacteria | Gammaproteobacteria | Pasteurellales | Pasteurellaceae | Actinobacillus | |
| OTU51 | Proteobacteria | Gammaproteobacteria | Vibrionales | Vibrionaceae | Aliivibrio | sifiae |
| OTU52 | Proteobacteria | Gammaproteobacteria | Vibrionales | Vibrionaceae | Aliivibrio | |
| OTU53 | Proteobacteria | Gammaproteobacteria | Vibrionales | Vibrionaceae | Aliivibrio | logei |
| OTU54 | Proteobacteria | Gammaproteobacteria | Vibrionales | Vibrionaceae | Aliivibrio | finisterrensis |
| OTU55 | Proteobacteria | Gammaproteobacteria | Vibrionales | Vibrionaceae | Aliivibrio | wodanis |
| OTU56 | Actinobacteria | Actinobacteria | Actinomycetales | Actinomycetaceae | Arcanobacterium | phocae |
| OTU57 | Actinobacteria | Actinobacteria | Actinomycetales | Actinomycetaceae | Arcanobacterium | phocisimile |
| OTU58 | Actinobacteria | Actinobacteria | Actinomycetales | Actinomycetaceae | Arcanobacterium | |
| OTU59 | Actinobacteria | Actinobacteria | Actinomycetales | Actinomycetaceae | Arcanobacterium | haemolyticum |
| OTU60 | Proteobacteria | Epsilonproteobacteria | Campylobacterales | Campylobacteraceae | Arcobacter | |
| OTU61 | Firmicutes | Bacilli | Lactobacillales | Carnobacteriaceae | Atopobacter | phocae |
| OTU62 | Firmicutes | Bacilli | Bacillales | Bacillaceae | Bacillus | decisifrondis |
| OTU63 | Bacteroidetes | Bacteroidia | Bacteroidales | Bacteroidaceae | Bacteroides | pyogenes |
| OTU64 | Bacteroidetes | Bacteroidia | Bacteroidales | Bacteroidaceae | Bacteroides | |
| OTU65 | Bacteroidetes | Flavobacteriia | Flavobacteriales | Flavobacteriaceae | Bergeyella | zoohelcum |
| OTU66 | Proteobacteria | Gammaproteobacteria | Pasteurellales | Pasteurellaceae | Bibersteinia | |
| OTU67 | Proteobacteria | Gammaproteobacteria | Pasteurellales | Pasteurellaceae | Bisgaardia | |
| OTU68 | Actinobacteria | Actinobacteria | Actinomycetales | Geodermatophilaceae | Blastococcus | saxobsidens |
| OTU69 | Actinobacteria | Actinobacteria | Actinomycetales | Dermabacteraceae | Brachybacterium | squillarum |

| | | | | | | |
|---|---|---|---|---|---|---|
| OTU70 | Actinobacteria | Actinobacteria | Actinomycetales | Dermabacteraceae | Brachybacterium | muris |
| OTU71 | Proteobacteria | Betaproteobacteria | Burkholderiales | Comamonadaceae | Brachymonas | |
| OTU72 | Proteobacteria | Betaproteobacteria | Burkholderiales | Burkholderiaceae | Burkholderia | |
| OTU73 | Proteobacteria | Betaproteobacteria | Burkholderiales | Burkholderiaceae | Burkholderia | multivorans |
| OTU74 | Proteobacteria | Epsilonproteobacteria | Campylobacterales | Campylobacteraceae | Campylobacter | |
| OTU75 | Proteobacteria | Gammaproteobacteria | Cardiobacteriales | Cardiobacteriaceae | Cardiobacterium | |
| OTU76 | Firmicutes | Clostridia | Clostridiales | Lachnospiraceae | Catonella | morbi |
| OTU77 | Proteobacteria | Alphaproteobacteria | Caulobacterales | Caulobacteraceae | Caulobacter | |
| OTU78 | Bacteroidetes | Flavobacteriia | Flavobacteriales | Flavobacteriaceae | Chryseobacterium | |
| OTU79 | Firmicutes | Clostridia | Clostridiales | Clostridiaceae | Clostridium | perfringens |
| OTU80 | Firmicutes | Clostridia | Clostridiales | Clostridiaceae | Clostridium | |
| OTU81 | Proteobacteria | Gammaproteobacteria | Alteromonadales | Colwelliaceae | Colwellia | aestuarii |
| OTU82 | Actinobacteria | Actinobacteria | Actinomycetales | Corynebacteriaceae | Corynebacterium | phocae |
| OTU83 | Actinobacteria | Actinobacteria | Actinomycetales | Corynebacteriaceae | Corynebacterium | |
| OTU84 | Actinobacteria | Actinobacteria | Actinomycetales | Corynebacteriaceae | Corynebacterium | amycolatum |
| OTU85 | Actinobacteria | Actinobacteria | Actinomycetales | Corynebacteriaceae | Corynebacterium | tuberculostearicum |
| OTU86 | Actinobacteria | Actinobacteria | Actinomycetales | Corynebacteriaceae | Corynebacterium | ulcerans |
| OTU87 | Proteobacteria | Betaproteobacteria | Burkholderiales | Comamonadaceae | Delftia | |
| OTU88 | Proteobacteria | Deltaproteobacteria | Desulfovibrionales | Desulfomicrobiaceae | Desulfomicrobium | |
| OTU89 | Proteobacteria | Deltaproteobacteria | Desulfovibrionales | Desulfomicrobiaceae | Desulfomicrobium | orale |
| OTU90 | Actinobacteria | Actinobacteria | Actinomycetales | Dietziaceae | Dietzia | |
| OTU91 | Actinobacteria | Actinobacteria | Actinomycetales | Dietziaceae | Dietzia | cinnamea |
| OTU92 | Actinobacteria | Actinobacteria | Actinomycetales | Dietziaceae | Dietzia | papillomatosis |
| OTU93 | Firmicutes | Clostridia | Clostridiales | Lachnospiraceae | Dorea | |
| OTU94 | Proteobacteria | Gammaproteobacteria | Pseudomonadales | Moraxellaceae | Enhydrobacter | aerosaccus |
| OTU95 | Firmicutes | Bacilli | Lactobacillales | Enterococcaceae | Enterococcus | |
| OTU96 | Firmicutes | Bacilli | Lactobacillales | Enterococcaceae | Enterococcus | faecium |
| OTU97 | Firmicutes | Clostridia | Clostridiales | Clostridiales Family XIII. Incertae Sedis | Eubacterium | sulci |
| OTU98 | Firmicutes | Clostridia | Clostridiales | Peptostreptococcaceae | Filifactor | villosus |
| OTU99 | Firmicutes | Clostridia | Clostridiales | Clostridiales Family XI. Incertae Sedis | Finegoldia | magna |
| OTU100 | Firmicutes | Clostridia | Clostridiales | Peptoniphilaceae | Finegoldia | magna |
| OTU101 | Firmicutes | Clostridia | Clostridiales | Peptoniphilaceae | Finegoldia | |
| OTU102 | Fusobacteria | Fusobacteriia | Fusobacteriales | Fusobacteriaceae | Fusobacterium | necrophorum |
| OTU103 | Fusobacteria | Fusobacteriia | Fusobacteriales | Fusobacteriaceae | Fusobacterium | |
| OTU104 | Fusobacteria | Fusobacteriia | Fusobacteriales | Fusobacteriaceae | Fusobacterium | mortiferum |

| | | | | | | |
|---|---|---|---|---|---|---|
| OTU105 | Fusobacteria | Fusobacteriia | Fusobacteriales | Fusobacteriaceae | Fusobacterium | canifelinum |
| OTU106 | Firmicutes | Bacilli | Lactobacillales | Aerococcaceae | Globicatella | |
| OTU107 | Actinobacteria | Actinobacteria | Actinomycetales | Gordoniaceae | Gordonia | |
| OTU108 | Firmicutes | Clostridia | Clostridiales | Clostridiales Family XI. Incertae Sedis | Helcococcus | ovis |
| OTU109 | Proteobacteria | Epsilonproteobacteria | Campylobacterales | Helicobacteraceae | Helicobacter | |
| OTU110 | Bacteroidetes | Sphingobacteriia | Sphingobacteriales | Chitinophagaceae | Hydrotalea | |
| OTU111 | Actinobacteria | Actinobacteria | Actinomycetales | Micrococcaceae | Kocuria | palustris |
| OTU112 | Firmicutes | Bacilli | Lactobacillales | Lactobacillaceae | Lactobacillus | iners |
| OTU113 | Proteobacteria | Gammaproteobacteria | Pasteurellales | Pasteurellaceae | Mannheimia | varigena |
| OTU114 | Proteobacteria | Gammaproteobacteria | Oceanospirillales | Oceanospirillaceae | Marinomonas | pontica |
| OTU115 | Proteobacteria | Gammaproteobacteria | Oceanospirillales | Oceanospirillaceae | Marinomonas | polaris |
| OTU116 | Proteobacteria | Gammaproteobacteria | Oceanospirillales | Oceanospirillaceae | Marinomonas | |
| OTU117 | Proteobacteria | Alphaproteobacteria | Rhizobiales | Methylobacteriaceae | Methylobacterium | |
| OTU118 | Proteobacteria | Betaproteobacteria | Methylophilales | Methylophilaceae | Methylotenera | |
| OTU119 | Actinobacteria | Actinobacteria | Actinomycetales | Microbacteriaceae | Microbacterium | ginsengisoli |
| OTU120 | Actinobacteria | Actinobacteria | Actinomycetales | Micrococcaceae | Micrococcus | |
| OTU121 | Actinobacteria | Actinobacteria | Actinomycetales | Micrococcaceae | Micrococcus | lylae |
| OTU122 | Actinobacteria | Actinobacteria | Actinomycetales | Micrococcaceae | Micrococcus | cohnii |
| OTU123 | Proteobacteria | Gammaproteobacteria | Pseudomonadales | Moraxellaceae | Moraxella | |
| OTU124 | Proteobacteria | Gammaproteobacteria | Pseudomonadales | Moraxellaceae | Moraxella | catarrhalis |
| OTU125 | Proteobacteria | Gammaproteobacteria | Pseudomonadales | Moraxellaceae | Moraxella | equi |
| OTU126 | Proteobacteria | Gammaproteobacteria | Enterobacteriales | Enterobacteriaceae | Morganella | morganii |
| OTU127 | Proteobacteria | Gammaproteobacteria | Alteromonadales | Moritellaceae | Moritella | |
| OTU128 | Tenericutes | Mollicutes | Mycoplasmatales | Mycoplasmataceae | Mycoplasma | |
| OTU129 | Tenericutes | Mollicutes | Mycoplasmatales | Mycoplasmataceae | Mycoplasma | phocidae |
| OTU130 | Proteobacteria | Betaproteobacteria | Neisseriales | Neisseriaceae | Neisseria | zoodegmatis |
| OTU131 | Proteobacteria | Betaproteobacteria | Neisseriales | Neisseriaceae | Neisseria | animaloris |
| OTU132 | Proteobacteria | Betaproteobacteria | Neisseriales | Neisseriaceae | Neisseria | |
| OTU133 | Actinobacteria | Actinobacteria | Actinomycetales | Nocardioidaceae | Nocardioides | |
| OTU134 | Proteobacteria | Gammaproteobacteria | Oceanospirillales | Oceanospirillaceae | Oleispira | antarctica |
| OTU135 | Proteobacteria | Gammaproteobacteria | Oceanospirillales | Oceanospirillaceae | Oleispira | |
| OTU136 | Bacteroidetes | Flavobacteriia | Flavobacteriales | Flavobacteriaceae | Ornithobacterium | rhinotracheale |
| OTU137 | Proteobacteria | Gammaproteobacteria | Pasteurellales | Pasteurellaceae | Otariodibacter | oris |
| OTU138 | Proteobacteria | Gammaproteobacteria | Pasteurellales | Pasteurellaceae | Otariodibacter | |
| OTU139 | Proteobacteria | Gammaproteobacteria | Pasteurellales | Pasteurellaceae | Pasteurella | multocida |

| | | | | | | |
|---|---|---|---|---|---|---|
| OTU140 | Proteobacteria | Gammaproteobacteria | Pasteurellales | Pasteurellaceae | Pasteurella | |
| OTU141 | Proteobacteria | Gammaproteobacteria | Pasteurellales | Pasteurellaceae | Pasteurella | oralis |
| OTU142 | Proteobacteria | Betaproteobacteria | Burkholderiales | Comamonadaceae | Pelomonas | puraquae |
| OTU143 | Firmicutes | Clostridia | Clostridiales | Peptococcaceae | Peptococcus | |
| OTU144 | Firmicutes | Clostridia | Clostridiales | Peptoniphilaceae | Peptoniphilus | |
| OTU145 | Proteobacteria | Alphaproteobacteria | Caulobacterales | Caulobacteraceae | Phenylobacterium | |
| OTU146 | Proteobacteria | Gammaproteobacteria | Vibrionales | Vibrionaceae | Photobacterium | damselae |
| OTU147 | Proteobacteria | Gammaproteobacteria | Vibrionales | Vibrionaceae | Photobacterium | |
| OTU148 | Proteobacteria | Gammaproteobacteria | Vibrionales | Vibrionaceae | Photobacterium | swingsii |
| OTU149 | Proteobacteria | Betaproteobacteria | Burkholderiales | Burkholderiaceae | Polynucleobacter | necessarius |
| OTU150 | Bacteroidetes | Bacteroidia | Bacteroidales | Porphyromonadaceae | Porphyromonas | gulae |
| OTU151 | Bacteroidetes | Bacteroidia | Bacteroidales | Porphyromonadaceae | Porphyromonas | |
| OTU152 | Bacteroidetes | Bacteroidia | Bacteroidales | Prevotellaceae | Prevotella | melaninogenica |
| OTU153 | Cyanobacteria | Prochlorales | Prochlorales | Prochlorococcaceae | Prochlorococcus | |
| OTU154 | Actinobacteria | Actinobacteria | Actinomycetales | Propionibacteriaceae | Propionibacterium | acnes |
| OTU155 | Actinobacteria | Actinobacteria | Actinomycetales | Propionibacteriaceae | Propionibacterium | |
| OTU156 | Proteobacteria | Gammaproteobacteria | Alteromonadales | Pseudoalteromonadaceae | Pseudoalteromonas | |
| OTU157 | Proteobacteria | Gammaproteobacteria | Alteromonadales | Pseudoalteromonadaceae | Pseudoalteromonas | marina |
| OTU158 | Proteobacteria | Gammaproteobacteria | Pseudomonadales | Pseudomonadaceae | Pseudomonas | |
| OTU159 | Proteobacteria | Gammaproteobacteria | Pseudomonadales | Pseudomonadaceae | Pseudomonas | xanthomarina |
| OTU160 | Proteobacteria | Gammaproteobacteria | Xanthomonadales | Xanthomonadaceae | Pseudomonas | |
| OTU161 | Proteobacteria | Gammaproteobacteria | Pseudomonadales | Moraxellaceae | Psychrobacter | aestuarii |
| OTU162 | Proteobacteria | Gammaproteobacteria | Pseudomonadales | Moraxellaceae | Psychrobacter | jeotgali |
| OTU163 | Proteobacteria | Gammaproteobacteria | Pseudomonadales | Moraxellaceae | Psychrobacter | |
| OTU164 | Proteobacteria | Gammaproteobacteria | Pseudomonadales | Moraxellaceae | Psychrobacter | sanguinis |
| OTU165 | Proteobacteria | Gammaproteobacteria | Pseudomonadales | Moraxellaceae | Psychrobacter | lutiphocae |
| OTU166 | Proteobacteria | Gammaproteobacteria | Pseudomonadales | Moraxellaceae | Psychrobacter | celer |
| OTU167 | Proteobacteria | Gammaproteobacteria | Pseudomonadales | Moraxellaceae | Psychrobacter | arenosus |
| OTU168 | Actinobacteria | Actinobacteria | Actinomycetales | Micrococcaceae | Rothia | mucilaginosa |
| OTU169 | Proteobacteria | Gammaproteobacteria | Alteromonadales | Shewanellaceae | Shewanella | |
| OTU170 | Firmicutes | Bacilli | Bacillales | Staphylococcaceae | Staphylococcus | schleiferi |
| OTU171 | Firmicutes | Bacilli | Bacillales | Staphylococcaceae | Staphylococcus | sciuri |
| OTU172 | Firmicutes | Bacilli | Bacillales | Staphylococcaceae | Staphylococcus | |
| OTU173 | Firmicutes | Bacilli | Bacillales | Staphylococcaceae | Staphylococcus | chromogenes |
| OTU174 | Firmicutes | Bacilli | Bacillales | Staphylococcaceae | Staphylococcus | hominis |

| | | | | | | |
|---|---|---|---|---|---|---|
| OTU175 | Firmicutes | Bacilli | Bacillales | Staphylococcaceae | Staphylococcus | warneri |
| OTU176 | Firmicutes | Bacilli | Bacillales | Staphylococcaceae | Staphylococcus | epidermidis |
| OTU177 | Firmicutes | Bacilli | Lactobacillales | Streptococcaceae | Streptococcus | dysgalactiae |
| OTU178 | Firmicutes | Bacilli | Lactobacillales | Streptococcaceae | Streptococcus | |
| OTU179 | Firmicutes | Bacilli | Lactobacillales | Streptococcaceae | Streptococcus | thermophilus |
| OTU180 | Firmicutes | Bacilli | Lactobacillales | Streptococcaceae | Streptococcus | phocae |
| OTU181 | Firmicutes | Bacilli | Lactobacillales | Streptococcaceae | Streptococcus | pseudopneumoniae |
| OTU182 | Firmicutes | Bacilli | Lactobacillales | Streptococcaceae | Streptococcus | suis |
| OTU183 | Proteobacteria | Gammaproteobacteria | Cardiobacteriales | Cardiobacteriaceae | Suttonella | indologenes |
| OTU184 | Bacteroidetes | Bacteroidia | Bacteroidales | Porphyromonadaceae | Tannerella | forsythia |
| OTU185 | Proteobacteria | Gammaproteobacteria | Alteromonadales | Colwelliaceae | Thalassomonas | agariperforans |
| OTU186 | Proteobacteria | Alphaproteobacteria | Rhizobiales | Phyllobacteriaceae | Thermovum | |
| OTU187 | Spirochaetes | Spirochaetia | Spirochaetales | Spirochaetaceae | Treponema | |
| OTU188 | Spirochaetes | Spirochaetia | Spirochaetales | Spirochaetaceae | Treponema | denticola |
| OTU189 | Spirochaetes | Spirochaetia | Spirochaetales | Spirochaetaceae | Treponema | putidum |
| OTU190 | Spirochaetes | Spirochaetia | Spirochaetales | Spirochaetaceae | Treponema | maltophilum |
| OTU191 | Spirochaetes | Spirochaetia | Spirochaetales | Spirochaetaceae | Treponema | medium |
| OTU192 | Tenericutes | Mollicutes | Mycoplasmatales | Mycoplasmataceae | Ureaplasma | |
| OTU193 | Firmicutes | Bacilli | Bacillales | Planococcaceae | Ureibacillus | terrenus |
| OTU194 | Proteobacteria | Gammaproteobacteria | Vibrionales | Vibrionaceae | Vibrio | |
| OTU195 | Proteobacteria | Gammaproteobacteria | Vibrionales | Vibrionaceae | Vibrio | parahaemolyticus |
| OTU196 | Proteobacteria | Gammaproteobacteria | Vibrionales | Vibrionaceae | Vibrio | cyclitrophicus |
| OTU197 | Proteobacteria | Gammaproteobacteria | Vibrionales | Vibrionaceae | Vibrio | vulnificus |
| OTU198 | Proteobacteria | Gammaproteobacteria | Vibrionales | Vibrionaceae | Vibrio | hangzhouensis |
| OTU199 | Proteobacteria | Gammaproteobacteria | Vibrionales | Vibrionaceae | Vibrio | penaeicida |
| OTU200 | Proteobacteria | Gammaproteobacteria | Vibrionales | Vibrionaceae | Vibrio | gallaecicus |
| OTU201 | Proteobacteria | Gammaproteobacteria | Vibrionales | Vibrionaceae | Vibrio | tapetis |
| OTU202 | Proteobacteria | Gammaproteobacteria | Vibrionales | Vibrionaceae | Vibrio | toranzoniae |
| OTU203 | Proteobacteria | Gammaproteobacteria | Vibrionales | Vibrionaceae | Vibrio | atypicus |
| OTU204 | Proteobacteria | Gammaproteobacteria | Vibrionales | Vibrionaceae | Vibrio | ponticus |
| OTU205 | Proteobacteria | Gammaproteobacteria | Vibrionales | Vibrionaceae | Vibrio | rotiferianus |
